# *Mos1*-Mediated Transgenesis to Probe Consequences of Single Gene Mutations in Variation-Rich Isolates of *Caenorhabditis elegans*

**DOI:** 10.1371/journal.pone.0048762

**Published:** 2012-11-14

**Authors:** Maja Tarailo-Graovac, Nansheng Chen

**Affiliations:** Department of Molecular Biology and Biochemistry, Simon Fraser University, Burnaby, British Columbia, Canada; Inserm U869, France

## Abstract

*Caenorhabditis elegans*, especially the N2 isolate, is an invaluable biological model system. Numerous additional natural *C. elegans* isolates have been shown to have unexpected genotypic and phenotypic variations which has encouraged researchers to use next generation sequencing methodology to develop a more complete picture of genotypic variations among the isolates. To understand the phenotypic effects of a genomic variation (GV) on a single gene, in a variation-rich genetic background, one should analyze that particular GV in a well understood genetic background. In *C. elegans*, the analysis is usually done in N2, which requires extensive crossing to bring in the GV. This can be a very time consuming procedure thus it is important to establish a fast and efficient approach to test the effect of GVs from different isolates in N2. Here we use a Mos1-mediated single-copy insertion (MosSCI) method for phenotypic assessments of GVs from the variation-rich Hawaiian strain CB4856 in N2. Specifically, we investigate effects of variations identified in the CB4856 strain on *tac-1* which is an essential gene that is necessary for mitotic spindle elongation and pronuclear migration. We show the usefulness of the MosSCI method by using EU1004 *tac-1(or402)* as a control. *or402* is a temperature sensitive lethal allele within a well-conserved TACC domain (transforming acidic coiled-coil) that results in a leucine to phenylalanine change at amino acid 229. CB4856 contains a variation that affects the second exon of *tac-1* causing a cysteine to tryptophan change at amino acid 94 also within the TACC domain. Using the MosSCI method, we analyze *tac-1* from CB4856 in the N2 background and demonstrate that the C94W change, albeit significant, does not cause any obvious decrease in viability. This MosSCI method has proven to be a rapid and efficient way to analyze GVs.

## Introduction

*C. elegans* is central to biomedical, molecular, cell and developmental biology research, and is among the best genetically and molecularly characterized and understood model organisms. The most studied and the best understood *C. elegans* strain is N2, which was obtained from mushroom compost in Bristol, England [1]. The genome of N2 was the first one of a multi-cellular animal that has been fully sequenced and published [2]. While N2 has been widely used in research as a model organism for the past 40 years [3] other *C. elegans* wild-strains have been isolated globally from human-associated habitats such as rotting fruits and compost heaps [3], [4]. With the goal of reaching a better understanding of genotypic and phenotypic differences between these strains, as well as studying the relationships between genetic interactions, the wild isolates have been subjected to either whole [5] or partial genome sequencing [4]. Genetic studies of different *C. elegans* wild-strains [4], [6], [7], [8], [9] have revealed little genetic diversity compared to closely related species [4], [7], [8], [9], [10], [11], [12], [13], [14], [15], [16], [17], yet comparable to genetic diversity among human populations [18].

CB4856, which was isolated in 1972 from a pineapple field in Hawaii [19], is the wild-isolate strain that has been most extensively compared to N2 both genetically and phenotypically. In addition to the large number of genome variations (small and large changes in DNA sequence) between N2 and CB4856 [20], [21], [22], [23], [24], a number of phenotypic differences between the strains have been described. For example, CB4856 contains multiple variations in a PAZ/PIWI domain-containing protein (*ppw-1*) which renders the Hawaiian strain resistant to effecting germline-expressed genes when feeding dsRNA directed against those genes [25]. Recently CB4856 was found to be resistant to avermectins due to a four-amino-acid deletion in the ligand-binding domain of GLC-1, the alpha-subunit of a glutamate-gated chloride channel [26]. Identification of this naturally occurring four-amino-acid deletion in GLC-1 of the Hawaiian strain is the first genetic evidence of a mechanism for nematode resistance to anthelmintics, this type of resistance, in many nematode species, represents a major global health and agricultural problem [26]. Genome variations (GVs) for other phenotypic differences between the two strains have been identified although some traits including egg-laying behaviour and vulva development do not have identified genetic bases. To identify a variation responsible for a particular phenotype in a variation-rich natural isolate like CB4856, one would typically introduce GVs into the N2 genetic background to follow the observable trait in order to map it to a chromosomal region. Alternatively, whole genome sequencing allows for a candidate gene approach in which a candidate variation for a particular trait is introduced into the well understood N2 background after extensive outcrossing and scored for a visible phenotype. However, the outcrossing procedure can be extremely time consuming and particularly difficult when the phenotypes are subtle or when the GVs cannot be followed using PCR or PCR followed by restriction enzyme digests. Overcoming these difficulties and establishing fast and efficient approaches for analyzing phenotypes of candidate GVs from the variation-rich strains in the N2 background is essential for analysis of phenotypic differences between the strains.

*Mos*1-mediated *s*ingle-*c*opy *i*nsertion (MosSCI) is a recently developed method in *C. elegans* that allows integration of transgenes as single copies at a defined genomic site [27]. MosSCI eliminates problems associated with common methods for generating transgenes in *C. elegans*, including concatenation of injected DNA, as well as formation of multicopy arrays that are overexpressed in somatic cells and silenced in the germline [27]. Furthermore genetically neutral Mos1 insertion alleles exist that allow expression of transgenes at endogenous levels [27], [28]. Recently we demonstrated a powerful aspect of the MosSCI method that allows us to increase gene copy number in a controlled fashion and analyze the consequence of doubling [28] or tripling [29] gene dosage on animal development in different genetic backgrounds. In this study we examined how genome variations affecting an essential gene from CB4856 behave in N2 using the MosSCI method. We examined GVs affecting an essential gene *tac-1* [30], [31], [32], [33]. We demonstrated the power of the method by phenocopying the lethal phenotype of *tac-1(or402)* from EU1004 strain [33]. Importantly, we showed that the non-synonymous radical change within the essential TACC domain does not cause an apparent decrease in viability in the N2 background. The protocol we describe here is fast and efficient for analysis of single-gene variations from variation-rich *C. elegans* strains in the extensively studied N2 background. Furthermore, this protocol is effective for ruling out lack of readily detectable phenotypes due to a presence of putative modifiers.

## Materials and Methods

### Strains and Culturing Conditions

The following mutant alleles were used in this work: *unc-119(ed3)*, *cxTi10882, or368/or402, ok3305, dpy-10(e128),* and *mT1*. The following strains were used in this work: N2 (Bristol strain as a wild-type), EU1004 [*tac-1(or402) II*], CB4856, EG6250 [*unc-119(ed3) III; cxTi10882 IV*] and VC2580 [*tac-1(ok3305)/mT1 II; +/mT1[dpy-10(e128)] III* ]. The alleles *dotSi120* and *dotSi121*, and JNC150 [*dotSi120 IV [Y54E2A.3^CB4856^ + unc-119(+)]*], JNC151 [(*tac-1(ok3305) II; dotSi120 IV [Y54E2A.3^CB4856^ + unc-119(+)]*], JNC152 [*dotSi121 IV [Y54E2A.3^EU1004^ + unc-119(+)]*] and JNC153 [*tac-1(ok3305) II; dotSi121 IV [Y54E2A.3^EU1004^ + unc-119(+)]*] strains were generated in this study. All strains were maintained using standard protocol on nematode growth media (NGM) plates seeded with OP50 bacteria [34]. Strains were maintained at 20°C while phenotypic analyses were performed at both 14°C and 25°C as noted in the manuscript.

### Mos1-mediated transgenesis

The *tac-1* locus was amplified using Phusion (NEB), high-fidelity DNA polymerase from either CB4856 or EU1004 single worm lysates. The following primers were used: forward- AAACTATTACCTTCGCCTTCGC and reverse-CTGGAAAATTGCAAGATTTTAATAG. Amplicons were cloned into the pCFJ178 vector, as described previously [27]. Similar to our previous findings using the essential cell cycle gene *cyb-3* [28], [29], we have also found that *tac-1*, when injected in high concentrations, results in a toxic effect. Thus to obtain stable single-copy insertions, we co-injected 5 ng/μl of *tac-1*-targetting constructs with 50 ng/μL of pJL43.1, 5 ng/μL pGH8, 5 ng/μL pCFJ104 and 2.5 ng/μL pCFJ90 into the gonad of 32 (*tac-1^CB4856^)* and 38 (*tac-1^EU1004^)* young adult P_0_ EG6250 hermaphrodites. The plates that contained wild-type looking *mCherry* expressing worms were starved at 25°C and then screened for stable integrants as previously described [27]. Within two weeks multiple stable lines were obtained for each construct. One of each was confirmed to contain a single, truncation-free, integration at the *cxTi10882* site. These were further analyzed as JNC150 (*tac-1^CB4856^)* and JNC152. (*tac-1^EU1004^*). Neither of these strains have any obvious increase in lethality or developmental delay when observed at 14°C, 20°C and 25°C (Table 1 and data not shown).

**Table 1:** Phenotypes of *tac-1* alleles.

Genotypes	Developmental arrests 14°C (%)	Developmental arrests 25°C (%)
N2 (reference)	0.8 (*n* = 1441)	1.4 (*n* = 1987)
*dotSi120 IV [Y54E2A.3^CB4856^ + unc-119(+)]*	0.6 (*n* = 1596)	1.2 (*n* = 1902)
*dotSi121 IV [Y54E2A.3^EU1004^ + unc-119(+)]*	0.4 (*n* = 1844)	1.5 (*n* = 1157)
EU1004 [*tac-1(or402) II*]	64.9 (*n* = 336)	94.9 (*n* = 295)
*tac-1(ok3305) II* (F_2_)	100 (*n* = 445)	100 (*n* = 394)
*tac-1(ok3305) II; dotSi120 IV [Y54E2A.3^CB4856^ + unc-119(+)]*	0.6 (*n* = 1647)	1.2 (*n* = 1714)
*tac-1(ok3305) II; dotSi121 IV [Y54E2A.3^EU1004^ + unc-119(+)]*	61.2 (*n* = 276)	94.4 (*n* = 697)

### Analysis of the *ok3305* knockout allele

*ok3305* is a 812bp deletion that removes the majority of the *tac-1* gene. Previously, the loss of TAC-1 was mainly studied using RNAi to deplete the *tac-1* product [30], [31], [32]. In the absence of TAC-1 progeny arrest as embryos due to defective microtubule formation [30], [31], [32]. The knockout allele, *tac-1(ok3305)*, also results in lethality and so it was kept balanced as a heterozygote over a translocation (*mT1*). However, the stage at which *tac-1(ok3305)* homozygotes arrest has not been determined previously. In this study, we analyzed VC2580 to determine *ok3305's* phenotype. We found that VC2580 segregates approximately 63% arrested embryos due to *mT1* translocation aneuploidies, ∼6% of *mT1* homozygotes are Dpy and sterile, ∼6% are wild-type looking *tac-1(ok3305)* progeny and 25% are *tac-1(ok3305)/mT1 II; +/mT1[dpy-10(e128)] III* heterozygotes. Analysis of the ∼6% of the *tac-1(ok3305)* homozygotes, segregated from *tac-1(ok3305)/mT1 II; +/mT1[dpy-10(e128)] III* heterozygous hermaphrodites, revealed that all *tac-1(ok3305)* homozygotes produce progeny of which 100% arrest as embryos (Table 1). This is a common phenotype for maternal effect genes. Namely, F_1_ *tac-1(ok3305)* homozygotes likely receive the TAC-1 protein from *tac-1(ok3305)/mT1 II; +/mT1[dpy-10(e128)]* heterozygous hermaphrodites that allows them to develop into adult animals. However, the F_2_ generation of *tac-1(ok3305)* homozygotes does not have any wild-type TAC-1, which leads to 100% embryonic arrest. This phenotype is similar to the phenotype observed when RNAi is used to deplete TAC-1 [30], [31], [32]. Thus, we conclude that TAC-1 is likely to be maternally supplied and that loss of TAC-1 results in maternal effect embryonic lethality.

### Use of the *ok3305* knockout allele in phenotypic analysis of *tac-1* GVs

First, we generated JNC150 and JNC152 males by heat shock. These males were then mated to *tac-1(ok3305)* homozygotes to generate JNC151 [(*tac-1(ok3305) II; dotSi120 IV [Y54E2A.3^CB4856^ + unc-119(+)]*] and JNC153 [*tac-1(ok3305) II; dotSi121 IV [Y54E2A.3^EU1004^ + unc-119(+)]*]. Unlike *tac-1(ok3305)* homozygotes, which arrest as embryos, JNC151 homozygotes are indistinguishable from N2 (Table 1), while JNC153 homozygotes are indistinguishable from EU1004 (Table 1).

### Phenotypic analysis

For each analysis, L4 hermaphrodites were grown on fresh OP50 plates at 14°C or 25°C. The hermaphrodites were transferred to fresh plates every 12 hours. Eggs that did not hatch were scored as embryonic arrest, while eggs that hatched but did not grow to adulthood were scored as larval arrest. Together, embryonic and larval arrest are reported as developmental arrest in Table 1.

## Results and Discussion

**Figure 1:**
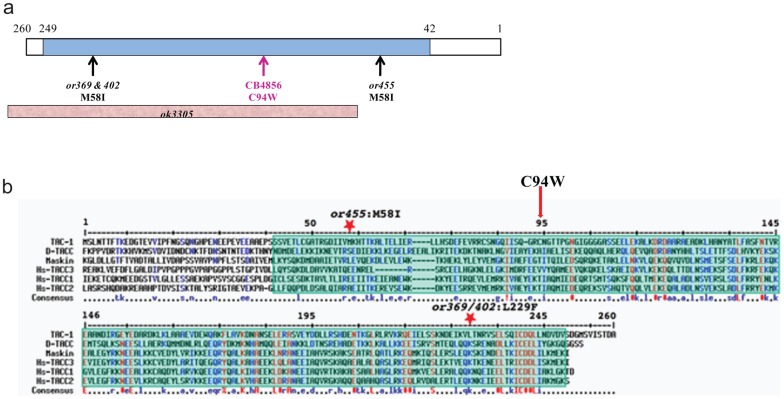
The C94W change is within the TACC domain. (**a**) Schematic representation of TAC-1 (1 to 260 amino-acid sequence). The majority of the protein is composed of the TACC domain which is depicted by the cyan box. Location and nature of all of the point mutants identified to date are shown as well. The *ok3305* knockout allele, which removes the majority of *tac-1* is depicted using a pink box. (**b**) The multiple sequence alignment of TAC-1 was adopted from Bellanger et al. 2007 [33]. The positions of the previously isolated point mutants *tac-1(or455)* and *tac-1(or369/402)* are depicted using red stars, while the C94W change identified in CB4856 is depicted using a red arrow. The cyan box highlights the presence of the TACC domain. All of the known point mutations occur within the TACC domain, but none of them affect conserved amino acids.

**Figure 2:**
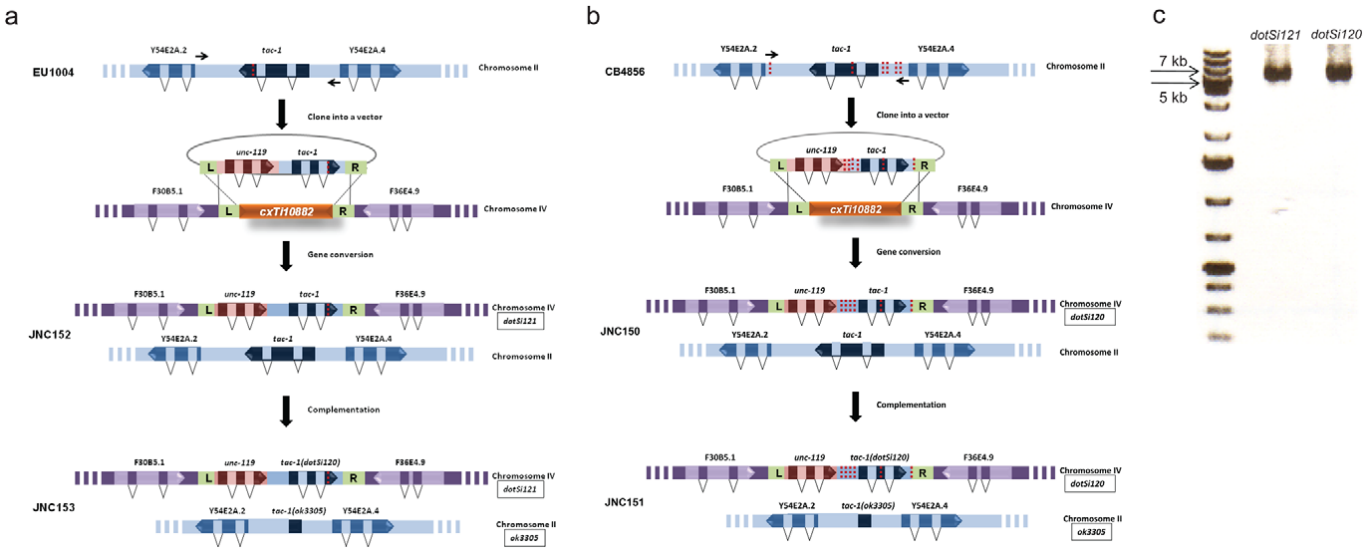
A single-copy transgene insertion used to investigate consequences of single gene mutations in variation-rich isolates of *C. elegans*. (**a**) Analysis of *tac-1(or402)* using *Mos1*-mediated transgenesis. *tac-1*, including its 5′ and 3′ regulatory sequences, was amplified using high-fidelity DNA polymerase from EU1004 genomic DNA and cloned into a pCFJ178 vector. The red dotted line located in the third exon of *tac-1* depicts the *or369/402* A to G change that results in an L229F amino acid change. Once cloned into the pCFJ178 vector, the transgene was inserted into the *cxTi10882* Mos1 (depicted in orange) integration site on chromosome IV (depicted in purple). The resulting JNC152 strain contains both the endogenous copy of *tac-1* located on chromosome II (depicted in blue), and *tac-1* isolated from EU1004 inserted on chromosome IV, *dotSi121*. To uncover the effect of *tac-1(or402)*, *dotSi121* was examined in the absence of endogenous TAC-1 using *ok3305*. Thus, we constructed JNC153. (**b**) Schematic representation of the method used to investigate consequences of *tac-1* variations detected in CB4856 *tac-1*, including the 5′ and 3′ regulatory sequences, was amplified using high-fidelity DNA polymerase from CB4856 genomic DNA and cloned into a pCFJ178 vector. The red dotted lines represent single nucleotide changes detected in CB4856 *tac-1*. Then, the transgene was inserted into the *cxTi10882* Mos1 (depicted in orange) integration site on chromosome IV (depicted in purple). JNC150 contains both the endogenous copy of *tac-1* located on chromosome II (depicted in blue) and *tac-1* isolated from CB4856 *dotSi120* inserted on chromosome IV. Then, *dotSi120* was analyzed in the absence of endogenous *tac-1(ok3305)*. (**c**) PCR bands of the expected size (6kb) for stably integrated single copy insertions of *tac-1*, *dotSi121* and *dotSi120*.

Recently we used whole-genome sequencing to identify GVs that disrupt protein-coding genes in CB4856 which is a wild-isolate strain of *C. elegans* from Hawaii (Vergara, Tarailo-Graovac, et al. in preparation). We were particularly interested in variations that are expected to cause a significant disruption of essential protein-coding genes. One such variation was identified within *tac-1* and we wanted to test the phenotype of this variation in the N2 background. *tac-1* is an essential gene and the only member of the transforming acidic coiled-coil (TACC) protein family in *C. elegans* whose function is crucial for pronuclear migration and mitotic spindle elongation [30], [31], [32]. In CB4856 we identified a number of single nucleotide changes that affect *tac-1*. In particular, one variation affected the second exon of *tac-1* causing a C94W change in the amino acid sequence (Figure 1a). To date, three point mutant alleles of *tac-1* have been isolated in genetic screens for temperature-sensitive mutants using EMS mutagenesis [33] in addition to one knockout allele. Both *tac-1(or369)* and *tac-1(or402)* have the same mutation in the TACC domain that results in a L229F amino acid change [33]. *tac-1(or455)* has a M58I change in the TACC domain [33]. Both of the amino acid changes occur within residues that are not highly conserved, yet the impact of these mutations is significant [33]. Both *tac-1(or369/or402)* and *tac-1(or455)* are temperature sensitive alleles and result in >95% embryonic arrest at a restrictive temperature [33]. Similar to these point mutations isolated in *tac-1*, the C94W change occurs in the essential TACC domain (Figure 1a) but also does not affect a highly conserved residue (Figure 1b). Since the C94W variation results in cysteine, which is polar amino acid, being replaced by the non-polar amino acid tryptophan, the C94W change would be considered more radical than either the L229F or the M58I changes, yet CB4856 animals do not have a temperature sensitive phenotype as the EMS-derived point mutants do. Thus, we were interested to see whether the lack of phenotype may be due to a presence of modifying mutations in CB4856 or simply because the C94W change does not affect TAC-1 function while the L229F and M58I changes do. Inspired by the enormous potential of the recently developed MosSCI method [27], which allowed us to show that doubling the dosage of the Cyclin B3 in *C. elegans* bypasses the need for the functional spindle-assembly checkpoint component MDF-1/Mad1 for survival beyond the third generation [28], [29], we decided to investigate an effect of GVs detected in CB4856 within *tac-1* in the N2 background using the MosSCI method (Figures 1 and 2).

The MosSCI method relies on the presence of a Mos1 insertion at the specific locus in *C. elegans* genome [27]. A large collection of mapped Mos1 insertion alleles has been generated by the European NEMAGENETAG consortium [35]. Many of the Mos1 insertions were identified as “genetically neutral” (inserted within genomic regions 3′ to coding genes) and shown to have robust germline expression [27], [36]. Previously, we successfully used the *ttTi5605* [28] and *cxTi10882* [29] Mos1 insertions, which are located at the center of chromosomes II and IV respectively, to study effects of *cyb-3* dosage on *C. elegans* development and anaphase onset [28], [29]. Neither of these Mos1 insertions interfered with the proper function of the inserted *cyb-3* gene [28], [29]. For the analysis of the GVs affecting *tac-1*, we selected the *cxTi10882* Mos1 insertion because it is located on different chromosome than *tac-1* [27], [36]. Since *cxTi10882* is located at the centre of chromosome IV and natural *tac-1* position is at the distal end of chromosome II, inserting *tac-1* into the *cxTi10882* Mos1 site should place it in a different genomic environment [37]. Namely, individual autosomes as well as chromosome arms and centers differ in several important properties including content of highly expressed genes, repetitive elements, and chromatin composition [37]. To test the *cxTi10882* Mos1 insertion site and the method we asked whether the temperature sensitive phenotype of *tac-1(or402)* allele could be phenocopied using this approach. We amplified *tac-1* including 5′ sequence immediately upstream of the predicted ATG initiator site and 3′ sequence immediately downstream of the predicted stop codon, from EU1004 (Figure 2a). Then, we cloned the *tac-1* amplicon into the pCFJ178 vector and inserted the transgene into the *cxTi10882* Mos1 integration site (Figure 2a). The strain that we generated contains an N2 copy of *tac-1* located at its endogenous position on chromosome II, as well as the chromosome IV integrated copy of EU1004 *tac-1* (*dotSi121*) that encodes the L229F change (Figure 2a). To uncover the effects of the L229F change, *tac-1* (*dotSi121*) must be analyzed in the absence of the endogenous *tac-1* gene product.

*tac-1(ok3305)* is an 812 bp deletion that removes the majority of *tac-1* (part of exon one, and exons two and three) and is likely to be a null mutation. To analyze *ok3305* we performed detailed phenotypic analysis (see MATERIALS AND METHODS). Our analysis revealed that *tac-1(ok3305)* results in maternal effect embryonic arrest. Namely, F_1_ *tac-1(ok3305)* homozygotes likely receive wild-type TAC-1 protein from *tac-1* heterozygous P_0_s that allows them to develop into adult animals. However, F_2_ homozygotes do not have any wild-type TAC-1, which leads to 100% of embryonic arrests (Table 1). The embryonic arrest phenotype is putatively identical to the phenotype observed when RNAi is used to deplete TAC-1 [30], [31], [32]. Thus, we conclude that *ok3305* is presumably a null allele of *tac-1*.

Next, we analyzed *tac-1* (*dotSi121*) in the absence of the endogenous *tac-1* product by using the *ok3305* knockout allele (Figure 2a). Our analysis revealed that the original strain EU1004 *tac-1(or368/or402) II* and the strain that we created using the MosSCI method JNC153 *tac-1(ok3305) II; dotSi121 IV [Y54E2A.3^EU1004^ + unc-119(+)]* are indistinguishable (Table 1). Phenocopying the lethal phenotype of the L229F change strongly supports the use of the outlined method for analysis of variations affecting a single gene.

Most heritable traits, including different susceptibility to disease and different responses to drug treatments, are genetically complex, resulting from contributions of mutations in many different genes [38]. Using model organisms, it has been shown that for the majority of phenotypes [39] and genes [40] the phenotypic consequence of identical GVs are affected by modifiers, variations present at other loci in the genome of the organism [41]. To determine whether the lack of phenotype may be due to a presence of modifiers in CB4856 or simply because the cysteine 94 residue is not essential for TAC-1 function, we analyzed CB4856 GVs affecting *tac-1* in the N2 background. In addition to the variation that results in the C94W amino acid change (Figure 1), *tac-1* has four mutations that are located upstream and one located downstream of the gene (Figure 2b). To rule out the possibility that these mutations may affect the expression of CB4856 *tac-1*, we investigated data from recent studies that analyzed differences in gene expression between N2 and CB4856 [42], [43]. Based on these data, *tac-1* does not appear to be differentially expressed in CB4865. Thus, to determine the effect of the C94W change in the N2 background, we decided to amplify CB4856 *tac-1*, including its 5′ and 3′ sequences. We cloned the amplicon into the pCFJ178 vector and inserted the transgene into the *cxTi10882* Mos1 integration site (Figure 2b). Then, we analyzed *tac-1* (*dotSi120*) that encodes the C94W change in the absence of endogenous *tac-1* gene product using the knockout allele (*ok3305*) (Figure 2b). Unlike L229F, C94W does not have any obvious effect on viability because 99.4% and 98.8% of the embryos analyzed at 14°C and 25°C respectively develop into adults, which is similar to what we have observed in N2 alone (Table 1). Thus, we were able to show, in a very time-effective manner, that variations affecting CB4856 *tac-1* do not result in an obvious phenotype that would be expected from a radical change, such as C94W, affecting the essential gene *tac-1*. The method outlined here has allowed us to very efficiently rule out the hypothesis that lack of a phenotypic consequence in the presence of C94W is due to putative GVs elsewhere in the CB4856 genome that modify the effects of the C94W variation. In contrast, these data suggest that L229 and M58 residues of the TACC domain are more sensitive to change than the C94 residue.

## Conclusion

Understanding the phenotypic consequence of GVs in different genetic backgrounds is of great importance for understanding phenotypic variations among individuals, especially disease susceptibility and treatment. To analyze the impact of GVs on a single gene and to test for the presence of putative modifiers due to natural variations, one needs to analyze causative variations in well understood and established genetic backgrounds. Here, we show a fast and efficient approach to analyzing GVs from variation-rich strains in the well-understood *C. elegans* N2 background. Using this approach, based on the MosSCI method, desired strains are usually generated within a few weeks. Alternative approaches that relay on extensive outcrossing usually take months. In addition to the time-consuming nature of the alternative approaches, the analysis may further be complicated by the involvement of other loci in variation rich-strains. For example, the analysis of CX11307 and JU751 wild-isolate strains for abamectin resistance, suggested the presence of a putative dominant resistance factor in addition to the *glc-1* variation [26]. Also, the analysis of resistance to dsRNA directed against germline-expressed genes [25] in CB4856 has suggested the presence of at least one other modifier allele in CB4856 in addition to the *ppw-1* mutation [25]. Furthermore, in the case of *tac-1*, an alternative analysis that relies on extensive outcrossing to place mutations in N2 would have been difficult due to the lack of obvious phenotypes and the fact that the C94W point mutation cannot be followed easily using PCR or even PCR followed by a restriction enzyme digest. Using the MosSCI method, we were able to show, in a very time-effective manner that the cysteine to tryptophan change in amino acid 94 of *tac-1* albeit significant, does not cause any decrease in viability in the N2 background. This result suggests that lack of phenotype CB4856 is not due to the presence of putative modifiers. Instead, cysteine at amino acid 94 may not play an important role for the function of the essential TACC domain and TAC-1. The usefulness of the approach that we describe in this manuscript also lies in its ability to be applied to engineer amino acid changes of interest rather than move already existing variations from one background to another. In such a way, one can analyze the effect of a specific amino acid on the development and viability of *C. elegans* by altering a particular amino acid. Furthermore, using this approach, genes from different *Caenorhabditis* and other species can be analyzed without having to deal with over-expression issues.
